# Population epidemiology and concordance for plasma amino acids and precursors in 11–12-year-old children and their parents

**DOI:** 10.1038/s41598-020-80923-9

**Published:** 2021-02-11

**Authors:** Stephanie Andraos, Katherine Lange, Susan A. Clifford, Beatrix Jones, Eric B. Thorstensen, Melissa Wake, David P. Burgner, Richard Saffery, Justin M. O’Sullivan

**Affiliations:** 1 Liggins Institute, The University of Auckland, Building 505, Level 2, 85 Park Road, Private Bag 92019, Auckland, 1142 New Zealand; 2 The Murdoch Children‘s Research Institute, Parkville, VIC 3052 Australia; 3 Department of Paediatrics, University of Melbourne, Parkville, VIC Australia; 4 Department of Statistics, Faculty of Science, The University of Auckland, Auckland, New Zealand; 5 Department of Paediatrics, Monash University, Clayton, VIC Australia; 6 MRC Lifecourse Epidemiology Unit, University of Southampton, Southampton, UK

**Keywords:** Metabolomics, Nutrition

## Abstract

Amino acid (AA) concentrations are influenced by both exogenous (e.g. diet, lifestyle) and endogenous factors (e.g. genetic, transcriptomic, epigenetic, and metabolomic). Fasting plasma AA profiles in adulthood are predictive of diabetes risk over periods of up to 12 years. Data on AA profiles in cross-generational cohorts, including individuals from shared gene-environment settings are scarce, but would allow the identification of the contribution of heritable and environmental factors characterising the levels of circulating AAs. This study aimed to investigate parent–child (familial dyad) concordance, absolute differences between generations- (children versus adults), age- (in adults: 28–71 years), and sex-dependent differences in plasma AA concentrations. Plasma AA concentrations were measured by UHPLC/MS–MS in 1166 children [mean (SD) age 11 (0.5) years, 51% female] and 1324 of their parents [44 (5.1) years, 87% female]. AA concentrations were variably concordant between parents and their children (5–41% of variability explained). Most AA concentrations were higher in adults than children, except for the non-essential AAs arginine, aspartic acid, glutamine, hydroxy-proline, proline, and serine. Male adults and children typically had higher AA concentrations than females. The exceptions were alanine, glutamine, glycine, hydroxy-proline, serine, and threonine in girls; and glycine and serine in women. Age, sex, and shared familial factors are important determinants of plasma AA concentrations.

## Introduction

Plasma amino acid (AA) concentrations are maintained under tight homeostatic control. Yet, changes in AA concentrations have been described in metabolic diseases^1,2^, asthma^3^, autism^4^, sepsis^5^, and malnutrition^6^. Moreover, fasting AA concentrations in middle-aged adults have been reported to predict diabetes onset 12 years later in the Framingham Offspring cohort^1^. Despite their clear role in health and disease, studies analyzing AA profiles in a shared family setting are scarce and those that exist are typically small in scale (e.g. 200 related individuals^7^), or only quantitate a limited selection of AAs^8^. Characterizing the epidemiology of plasma AAs and their familial concordance is of utmost importance given that AA profiles may predict disease^1^, and reflect both dietary and endogenous factors (i.e. genetic, transcriptomic, epigenetic and metabolic)^9,10^.

We previously identified strong familial concordance, sex and inter-generational differences, in the plasma concentrations of nutritional metabolites (e.g. Trimethylamine *N*-oxide (TMAO) and its precursors) in children and adults^8^. These data implicated gene-environment interactions in the setting and/or maintenance of metabolite concentrations. It may be possible to discern the relative gene/environment contributions to AA concentrations using the fact that some are solely diet-derived (essential), while others can also be synthesized de novo (non-essential)^9^.

In this study, we characterized AA concentrations in 1,166 children (51% females), and 1324 adults (87% females) from the CheckPoint study of Australian children and adults. We analyzed: (a) parent–child (familial dyad) concordance; (b) absolute differences between generations (adults versus children); (c) age as a continuous variable in the adult group (Mean (SD)) age: 44 (5) years; range 28–71 years in the adult subgroup); and (d) sex-specific effects on individual AA concentrations.

## Methods

### Ethical approval, consent, and sample collection

The study was approved by The Royal Children’s Hospital (Melbourne, Australia) Human Research Ethics Committee (33225D) and the Australian Institute of Family Studies Ethics Committee and was conducted in accordance with The Declaration of Helsinki. 1874 parent–child dyads participated in a biomedical assessment: The Child Health CheckPoint (CheckPoint), nested between waves 6 and 7 of the Longitudinal Study of Australian Children’s B cohort (LSAC)^11^. Parents or caregivers provided informed consent for themselves and their child to participate in the study and for the collection of their blood samples^12^ (Supplementary Fig. 1).

### Procedures and UHPLC/MS–MS analysis

Adults and children were semi-fasted. Mean (SD) fasting time was 4.4 (2.1) hours in children, and 3.4 (2.4) h in adults. Venous blood was collected from children and adults in EDTA tubes from single venepuncture split to components including 6 plasma aliquots (used for UHPLC/MS–MS analysis) processed within ~ 1 h (1 min to 3.8 h) prior to storage at − 80 °C^12^. A total of 2490 EDTA plasma samples were shipped on dry ice in thermally monitored boxes. Samples were then randomised as received from Melbourne on dry ice onto 34 different 96-well FluidX plates (Phenomenex), keeping parent–child pairs (1121 pairs) together on the same plate, and stored at – 80 °C prior to UHPLC/MS–MS analysis.

All AAs were measured using a Vanquish UHPLC + system, coupled with a TSQ Quantiva triple quadrupole mass spectrometer (Thermo Scientific) using a heated electrospray ionisation source (H-ESI) in positive ionization mode. Sample preparation was automated on an Eppendorf robot fitted with a thermal mixer and a vacuum manifold (EpMotion 5075vt, Germany). The UHPLC/MS–MS analysis and robotic automation has been described in detail elsewhere^13^. Briefly, protein precipitation was conducted by adding 300 µL of 1% formic acid in LC-Grade MeOH to 100 µL of either: (a) calibration curve standards, (b) plasma samples, (b) MilliQ H_2_O blanks, or (c) stripped plasma quadruplicate quality controls (QCs), at 3 different locations; all in a 96-well IMPACT protein precipitation plate (Phenomenex). 20 µL of an internal standard solution was added to all wells, the plate was capped, mixed (5 min, 800 rpm, room temperature), and the filtrate obtained by vacuum (450 mbar, 10 min). Tris (2-carboxyethyl) phosphine (100 µL, TCEP) was added for disulphide bond reduction. The reduced filtrate was agitated (15 min, 800 rpm, room temperature), and diluted with 200 µL of 1% ascorbic acid in MilliQ H_2_O. A Kinetex EVO C18 100 Å 150 × 2.1 mm 1.7 µm column (Phenomenex) at 40 °C, coupled with a Krudkatcher (Phenomenex) pre-column filter, was used to chromatographically separate the compounds. A flow of 400 µL/min starting at 2% acetonitrile and 98% mobile phase consisting of 5 mM perfluorohexanoic acid (PFHA) in MilliQ H_2_O was applied to the column, compounds of interest were eluted using an increasing acetonitrile gradient. The sample injection volume was 7 µL, and the run time was 15.5 min. All quality controls passed the acceptable cut-off for compound recovery and reproducibility, and QC results have been reported in detail elsewhere^13^.

### Statistical analysis

All statistical analyses were performed in R programming environment version 3.6.1^14^. Technical plate effects were removed from all metabolites using the *RANEF* function (*lme4* package in R)^15^. The reported AAs included essential AAs (i.e. valine, leucine, isoleucine, methionine, threonine, phenylalanine, and tryptophan), non-essential AAs (i.e. alanine, glycine, cysteine, serine, tyrosine, proline, histidine, arginine, asparagine, aspartic acid, glutamic acid, glutamine, taurine and citrulline), AA precursors (i.e. aminoadipic acid), and derivatives: methylated histidine (i.e. 1 and 3-methylhistidine), hydroxylated proline (i.e. OH-Proline), and adenylated methionine (i.e. *S*-Adenosylmethionine). Chromatographic issues occurred with lysine, cystathionine, and ornithine. Additionally, plasma concentrations of ethanolamine, homocysteine, and *S*-adenosylhomocysteine (SAH) were below the lowest limit of quantitation (LOQ) for most of our plates. These AAs were therefore excluded from our study.

Histograms of all plate-adjusted variables were plotted to assess normality.

3-Methylhistidine, aspartic acid, isoleucine, methionine, OH-proline, proline, and taurine were positively skewed and therefore log-transformed. The remaining AAs were normally distributed.

Two sets of mixed models were developed to test the effect of (a) family (shared gene-environment setting), and (b) generation (adults versus children) using the *lme4* package in R after adjusting for plate effects^15^. Log likelihoods were compared between models that contained both family (as a random effect) and generation (as a fixed effect), and those excluding one or the other. Pearson’s correlations adjusted for multiple testing using the *Holm* method in R were also conducted within parent–child dyads to confirm familial concordance. Family effect sizes were calculated as the ratio of the estimated family variance component divided by the total variance of each plate-adjusted variable.

Two sets of linear models for (a) sex, and (b) age (in the adult subgroup of 28–71 years) were also fitted for each plate-adjusted/log-transformed variable in children and adults separately. Given the narrow age distribution in children (11–12 years), we only characterized age-specific differences within the adults (28–71 years on a continuous scale).

## Results

### Sample characteristics and amino acid measurement

The CheckPoint cohort consisted of 2490 participants (1121 parent–child pairs); 1166 children (51% females), and 1324 adults (87% females, predominantly the children’s biological mothers) (Table 1).

**Table 1:** Sample characteristics.

Characteristic	Children			Adults		
	All	Male	Female	All	Male	Female
N	1166	565	601	1324	174	1150
Age in years [mean (SD)]	11.4 (0.5)	11.4 (0.5)	11.5 (0.5)	43.9 (5.1)	46.2 (6.4)	43.6 (4.8)
Biological parent of child (N)	N/A	N/A	N/A	1313	172	1141
BMI rounded in kg/m^2^ Median (Lower–Upper Quartiles)	18.4 (16.8–20.6)	18.1 (16.7–20.2)	18.8 (17.0–21.1)	26.54 (23.4–31.0)	27.4 (25.2–31.1)	26.3 (23.1–31.0)
BMI Z-scores [mean (SD)]	0.31 (0.9)	0.31 (0.9)	0.31 (0.9)	N/A	N/A	N/A
Australian state of current residence: State (N)	New South Wales (359); Victoria (261); Queensland (221); South Australia (92); West Australia (139); Tasmania (40); Northern Territory (17); Australian Capital Territory (38)			New South Wales (391); Victoria (311); Queensland (240); South Australia (108); West Australia (164); Tasmania (46); Northern Territory (18); Australian Capital Territory (47)		
Socio-Economic Indexes for Areas (SEIFA) disadvantage Quintile (N)	Most disadvantaged (83); second most (171); middle (199); second least (272); least disadvantaged (442)			Most disadvantaged (94); second most (193); middle (233); second least (304); least disadvantaged (501)		

Skewed variables were reported as medians and lower/upper quartiles, and normally distributed variables as means and standard deviations.

### Amino acid profiles are concordant between parents and children

**Figure 1:**
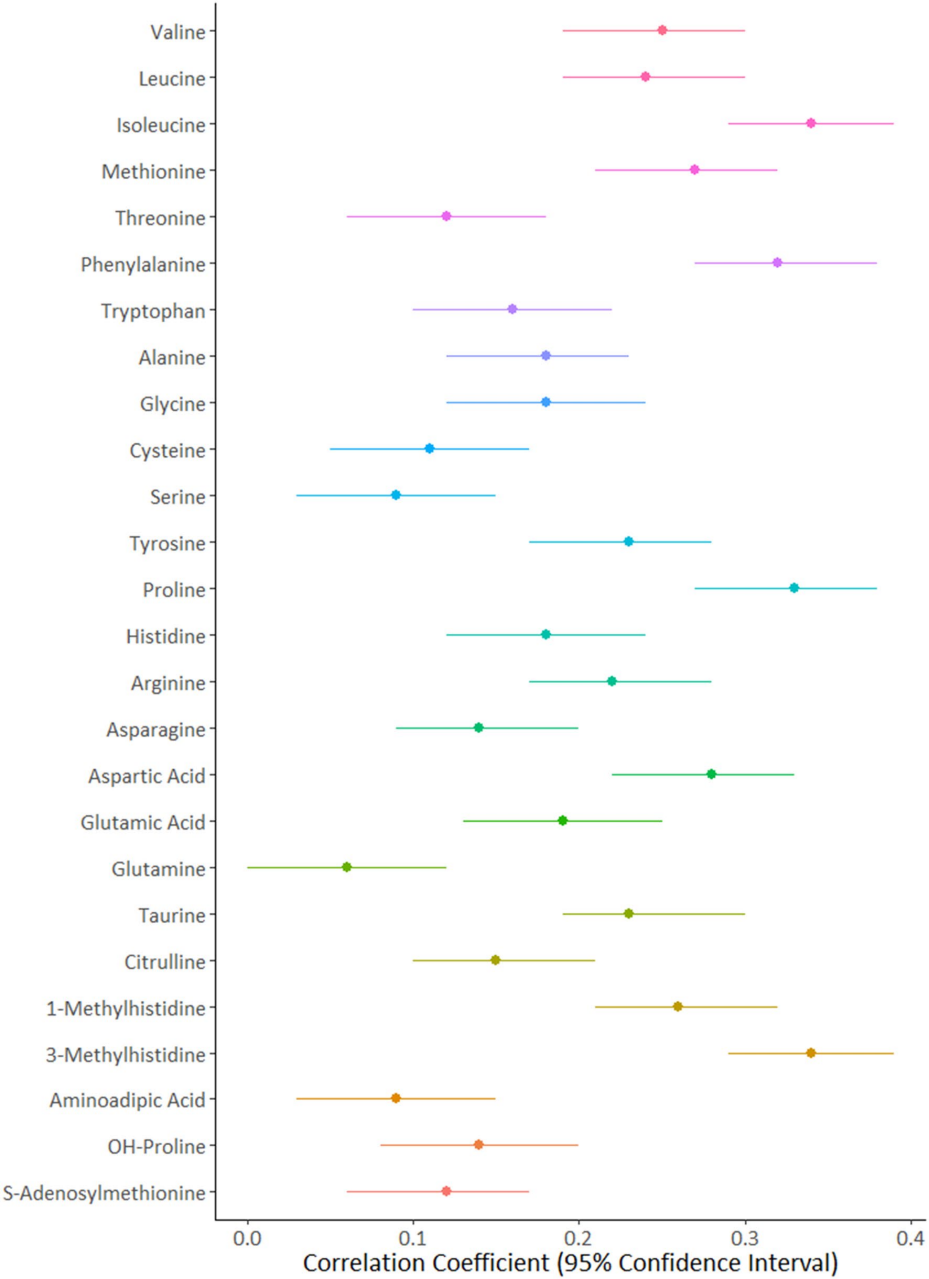
Forest plot of Pearson’s correlations within parent–child pairs.

All AA concentrations exhibited a concordance between children and parents from the same family (Table 2, Fig. 1, and Supplementary Figs. 2, 3, and 4). Both likelihood ratio tests and Pearson’s correlations between dyads showed the strongest familial effect to be for 3-methylhistidine (coefficient of correlation R = 0.34; confidence interval [0.29, 0.39]), and isoleucine (R = 0.34; [0.29, 0.39]), and the weakest for glutamine (R = 0.06; [0, 0.12]) and aminoadipic acid (R = 0.09 [0.03, 0.15]). In total, familial effects accounted for approximately 5% of variability in the dataset for glutamine and aminoadipic acid, 34% for isoleucine, and 41% for 3-methylhistidine (Supplementary Figs. 2, 3, and 4).

**Table 2:** Family effects, variances and effect sizes on amino acid levels.

Amino acid category	Amino acid^a^	Effect of Dyad (family) on mixed model	Family effect variance	Compound variance	Effect size of family on compound levels (%)^c^
Essential	Valine	p < 0.0001 Log likelihood with family = − 13,238 Log likelihood without family = − 13,272	615.30	2533.72	24
	Leucine	p < 0.0001 Log likelihood with family = − 12,069 Log likelihood without family = − 12,102	240.00	985.77	24
	Isoleucine^b^	p < 0.0001 Log likelihood with family = − 544 Log likelihood without family = − 614	0.03	0.10	34
	Methionine^b^	p < 0.0001 Log likelihood with family = − 185 Log likelihood without family = − 227	0.02	0.07	27
	Threonine	p < 0.0001 Log likelihood with family = − 11,747 Log likelihood without family = − 11,755	92.89	741.86	12
	Phenylalanine	p < 0.0001 Log likelihood with family = − 9509 Log likelihood without family = − 9571	41.52	131.37	32
	Tryptophan	p < 0.0001 Log likelihood with family = − 9955 Log likelihood without family = − 9969	32.59	175.91	18
Non- essential	Alanine	p < 0.0001 Log likelihood with family = − 14,335 Log likelihood without family = − 14,353	1027.00	5956.88	17
	Glycine	p < 0.0001 Log likelihood with family = − 13,658 Log likelihood without family = − 13,673	628.10	3463.75	18
	Cysteine	p = 0.0004 Log likelihood with family = − 10,209 Log likelihood without family = − 10,215	23.16	248.72	9
	Serine	p = 0.002 Log likelihood with family = − 11,283 Log likelihood without family = − 11,287	46.57	520.78	9
	Tyrosine	p < 0.0001 Log likelihood with family = − 10,435 Log likelihood without family = − 10,464	59.72	262.02	23
	Proline^b^	p < 0.0001 Log likelihood with family = − 636 Log likelihood without family = − 699	0.03	0.10	33
	Histidine	p < 0.0001 Log likelihood with family = − 10,259 Log likelihood without family = − 10,278	48.10	225.46	21
	Arginine	p < 0.0001 Log likelihood with family = − 10,726 Log likelihood without family = − 10,754	74.83	331.79	22
	Asparagine	p < 0.0001 Log likelihood with family = − 8877 Log likelihood without family = − 8889	10.10	74.10	14
	Aspartic acid^b^	p < 0.0001 Log likelihood with family = − 2309 Log likelihood without family = − 2350	0.19	0.55	36
	Glutamic acid	p < 0.0001 Log likelihood with family = − 10,507 Log likelihood without family = − 10,528	51.87	275.80	19
	Glutamine	p = 0.05 Log likelihood with family = − 15,450 Log likelihood without family = − 15,452	778.20	14,488.96	5
	Taurine^b^	p < 0.0001 Log likelihood with family = − 1489 Log likelihood without family = − 1524	0.05	0.20	25
	Citrulline	p < 0.0001 Log likelihood with family = − 8332 Log likelihood without family = − 8345	7.38	47.79	15
Precursors/derivatives	1-Methylhistidine	p < 0.0001 Log likelihood with family = − 3692 Log likelihood without family = − 3731	0.31	1.26	25
	3-Methylhistidine^b^	p < 0.0001 Log likelihood with family = − 2923 Log likelihood without family = − 2986	0.29	0.77	41
	Aminoadipic acid	p = 0.04 Log likelihood with family = − 2025 Log likelihood without family = − 2027	0.02	0.30	6
	OH-Proline^b^	p < 0.0001 Log likelihood with family = − 1905 Log likelihood without family = − 1913	0.03	0.46	7
	*S*-Adenosyl-methionine	p < 0.0001 Log likelihood with family = − 12,046 Log likelihood without family = − 12,054	119.0	941.72	13

^a^All in µM except for *S*-Adenosylmethionine in nM.

^b^Log transformed variables with back-transformed means and standard deviations.

^c^Calculated as the family (dyad) effect variance divided by the plate-adjusted compound variance × 100.

### AA profiles are age-dependent

Most AA concentrations exhibited differences between adults and children (p < 0.05), except for histidine, glutamic acid, isoleucine, tryptophan, tyrosine, and taurine (Table 3). Most AA concentrations were higher in adults, except for non-essential AAs and their derivatives: arginine (Mean (SD): 64.9 (19.1) µM in adults; 67.3 (17.1) µM in children), aspartic acid (3.8 (3.0) µM in adults; 4.43 (2.8) µM in children), glutamine (805.0 (125.0) µM in adults; 826.0 (114.0) µM in children), OH-proline (7.7 (2.2) µM in adults; 17.4 (1.5) µM in children), proline (192.0 (1.4) µM in adults; 197.0 (1.4) µM in children), and serine (90.4 (23.9) µM in adults; 97.8 (20.9) µM in children).

**Table 3:** Means, standard deviations (SDs) and generation effects of amino acids Table 3: Means, standard deviations (SDs) and generation effects of amino acids.

Amino acid category	Amino acid^a^	Parent mean (SD)	Child mean (SD)	Effect of generation on mixed model (children, adults)
Essential	Valine	216.0 (52.0)	204.0 (47.6)	p < 0.0001 Log likelihood with generation = − 13,238 Log likelihood without generation = − 13,261
	Leucine	114.0 (33.2)	108.0 (28.8)	p < 0.0001 Log likelihood with generation = − 12,069 Log likelihood without generation = − 12,086
	Isoleucine^b^	53.0 (1.4)	52.9 (1.4)	p = 0.20 Log likelihood with generation = − 543 Log likelihood without generation = − 544
	Methionine^b^	21.4 (1.3)	20.6 (1.3)	p < 0.0001 Log likelihood with generation = − 185 Log likelihood without generation = − 193
	Threonine	105.0 (29.2)	101.0 (24.7)	p = 0.0001 Log likelihood with generation = − 11,747 Log likelihood without generation = − 11,754
	Phenylalanine	57.2 (11.4)	53.4 (11.2)	p < 0.0001 Log likelihood with generation = − 9509 Log likelihood without generation = − 9558
	Tryptophan	48.9 (13.8)	48.9 (12.7)	p = 0.99 Log likelihood with generation = − 9955 Log likelihood without generation = − 9955
Non-essential	Alanine	307.0 (75.8)	302.0 (78.6)	p = 0.06 Log likelihood with generation = − 14,335 Log likelihood without generation = − 14,337
	Glycine	191.0 (70.3)	183.0 (41.8)	p = 0.0001 Log likelihood with generation = − 13,658 Log likelihood without generation = − 13,666
	Cysteine	78.1 (16.2)	66.3 (12.6)	p < 0.0001 Log likelihood with generation = − 10,209 Log likelihood without generation = − 10,400
	Serine	90.4 (23.9)	97.8 (20.9)	p < 0.0001 Log likelihood with generation = − 11,283 Log likelihood without generation = − 11,318
	Tyrosine	59.7 (16.7)	60.6 (15.6)	p = 0.13 Log likelihood with generation = − 10,435 Log likelihood without generation = − 10,436
	Proline^b^	192.0 (1.4)	197.0 (1.4)	p = 0.05 Log likelihood with generation = − 636 Log likelihood without generation = − 638
	Histidine	76.9 (16.4)	76.3 (13.2)	p = 0.21 Log likelihood with generation = − 10,259 Log likelihood without generation = − 10,260
	Arginine	64.9 (19.1)	67.3 (17.1)	P < 0.001 Log likelihood with generation = − 10,726 Log likelihood without generation = 10,732
	Asparagine	35.6 (8.5)	34.60 (8.7)	p = 0.003 Log likelihood with generation = − 8877 Log likelihood without generation = − 8882
	Aspartic acid^b^	3.8 (3.0)	4.43 (2.8)	p < 0.0001 Log likelihood with generation = − 2309 Log likelihood without generation = − 2318
	Glutamic acid	43.7 (17.0)	42.7 (16.1)	p = 0.11 Log likelihood with generation = − 10,507 Log likelihood without generation = − 10,508
	Glutamine	805.0 (125.0)	826.0 (114.0)	p < 0.0001 Log likelihood with generation = − 15,450 Log likelihood without generation = − 15,460
	Taurine^b^	73.4 (1.9)	78.6 (2.0)	p = 0.61 Log likelihood with generation = − 1489 Log likelihood without generation = − 1489
	Citrulline	26.4 (7.5)	26.0 (6.1)	p = 0.02 Log likelihood with generation = − 8332 Log likelihood without generation = − 8334
Precursors/derivatives	1-MethylHistidine	5.2 (1.2)	4.6 (1.0)	p < 0.0001 Log likelihood with generation = − 3692 Log likelihood without generation = − 3808
	3-MethylHistidine^b^	7.4 (2.6)	6.1 (2.6)	p < 0.0001 Log likelihood with generation = − 2923 Log likelihood without generation = − 2950
	Aminoadipic Acid	2.4 (0.7)	2.3 (0.3)	p < 0.0001 Log likelihood with generation = − 2025 Log likelihood without generation = − 2044
	OH-Proline^b^	7.7 (2.2)	17.4 (1.5)	p < 0.0001 Log likelihood with generation = − 1905 Log likelihood without generation = − 2522
	*S*-Adenosylmethionine	97.5 (32.2)	93.9 (28.8)	p = 0.002 Log likelihood with generation = − 12,046 Log likelihood without generation = − 12,051

^a^All in µM except for *S*-Adenosylmethionine in nM.

^b^Log transformed variables with back-transformed means and standard deviations.

Concentrations of 1-methylhistidine (adjusted R^2^ of the linear model = 0.01; p = 0.003), citrulline (R^2^ = 0.03; p < 0.0001), glutamic acid (R^2^ = 0.005; p < 0.01), glutamine (R^2^ = 0.01; p < 0.001), phenylalanine (R^2^ = 0.004; p = 0.01), *S*-adenosylmethionine (R^2^ = 0.02; p < 0.0001), and tyrosine (R^2^ = 0.01; p < 0.001) were all weakly positively associated with increasing age in the adult subgroup. By contrast, threonine concentrations (R^2^ = 0.002; p = 0.05) exhibited a weak negative association with adult age (Supplementary Table 1).

### Amino acid profiles are sex-dependent

Males had significantly higher concentrations for most plasma AAs in both adults and children (p < 0.05) (Table 4). Exceptions, where concentrations were higher in female children, were evident for the essential AA threonine [106.0 (25.2) µM in female children; 97.1 (23.4) µM in male children], and non-essential AAs alanine [307.0 (77.7) µM in female children; 296.0 (79.3) in male children], glutamine [839.0 (105.0) µM in female children ; 812.0 (121.0) µM in male children], glycine [189.0 (42.3) µM in female children; 176.0 (40.2) µM in male children], and serine [99.8 (21.3) µM in female children ; 95.7 (20.3) µM in male children]. Similarly, the AA derivative OH-proline was present at higher concentrations in female [18.1 (1.4) µM] than male children [16.7 (1.6) µM]. Adult females also exhibited higher plasma concentrations for non-essential AAs glycine [195.0 (72.8) µM in female adults; 165.0 (42.7) µM in male adults] and serine [91.5 (24.3) µM in female adults; 83.2 (18.8) µM in male adults].

**Table 4:** Means, standard deviations, and linear model results by sex and generation.

Amino acid category	Amino acid^a^	Children				Adults			
		Mean females (SD)	Mean males (SD)	Adjusted R^2^ of linear model	p value	Mean females (SD)	Mean males (SD)	Adjusted R^2^ of linear model	p value
Essential	Valine	200.0 (44.9)	209.0 (50.0)	0.01	< 0.001	211.0 (50.7)	251.0 (46.8)	0.06	< 0.0001
	Leucine	104.0 (26.9)	111.0 (30.5)	0.01	0.0001	111.0 (32.2)	136.0 (30.8)	0.07	< 0.0001
	Isoleucine^b^	51.8 (1.4)	54.0 (1.4)	0.003	0.02	51.5 (1.4)	63.9 (1.3)	0.05	< 0.0001
	Methionine^b^	20.5 (1.3)	20.8 (1.3)	0.0003	0.25	21.1 (1.3)	23.6 (1.3)	0.02	< 0.0001
	Threonine	106.0 (25.2)	97.1 (23.4)	0.03	< 0.0001	106.0 (30.0)	104.0 (23.1)	− 0.0003	0.47
	Phenylalanine	52.4 (10.4)	54.4 (11.9)	0.01	0.002	56.8 (11.2)	59.9 (12.2)	0.01	0.001
	Tryptophan	47.5 (12.0)	50.5 (13.2)	0.01	< 0.0001	48.4 (14.0)	52.5 (11.9)	0.01	0.0002
Non-essential	Alanine	307.0 (77.7)	296.0 (79.3)	0.004	0.02	304.0 (75.7)	328.0 (73.7)	0.01	0.0001
	Glycine	189.0 (42.3)	176.0 (40.2)	0.02	< 0.0001	195.0 (72.8)	165.0 (42.7)	0.02	< 0.0001
	Cysteine	65.1 (12.0)	67.6 (13.2)	0.01	< 0.001	76.7 (15.8)	87.6 (15.7)	0.05	< 0.0001
	Serine	99.8 (21.3)	95.7 (20.3)	0.01	0.001	91.5 (24.3)	83.2 (18.8)	0.01	< 0.0001
	Tyrosine	60.9 (15.0)	60.4 (16.3)	− 0.001	0.60	59.0 (16.8)	64.1 (15.1)	0.01	0.0002
	Proline^b^	198.0 (1.4)	196.0 (1.4)	− 0.0005	0.52	188.0 (1.4)	229.0 (1.3)	0.04	< 0.0001
	Histidine	76.8 (12.8)	75.7 (13.6)	0.001	0.19	77.1 (16.7)	76.3 (14.9)	− 0.0005	0.55
	Arginine	67.3 (17.2)	67.3 (17.0)	− 0.001	0.93	64.4 (19.2)	67.9 (17.6)	0.003	0.02
	Asparagine	34.1 (7.8)	35.2 (9.6)	0.003	0.04	35.7 (8.7)	35.1 (7.4)	− 0.0003	0.42
	Aspartic acid^b^	4.5 (2.8)	4.4 (2.7)	− 0.0005	0.51	3.7 (3.1)	4.2 (2.7)	0.002	0.06
	Glutamic acid	42.7 (16.9)	42.6 (15.3)	− 0.0008	0.91	41.8 (15.9)	56.4 (18.8)	0.08	< 0.0001
	Glutamine	839.0 (105.0)	812.0 (121.0)	0.01	< 0.0001	797.0 (124.0)	860.0 (121.0)	0.03	< 0.0001
	Taurine^b^	77.7 (2.0)	79.6 (1.9)	− 0.001	0.94	73.3 (1.9)	74.0 (2.0)	− 0.0005	0.55
	Citrulline	25.4 (5.9)	26.3 (6.3)	0.004	0.02	25.9 (7.4)	30.1 (7.2)	0.035	< 0.0001
Precursors/derivatives	1-Methylhistidine	4.6 (1.0)	4.6 (1.0)	− 0.001	0.69	5.1 (1.1)	6.1 (1.3)	0.08	< 0.0001
	3-Methylhistidine^b^	5.7 (2.7)	6.5 (2.5)	0.005	0.01	7.2 (2.7)	8.8 (2.2)	0.004	0.01
	Aminoadipic Acid	2.2 (0.2)	2.3 (0.3)	0.01	0.001	2.4 (0.7)	2.6 (0.4)	0.01	< 0.0001
	OH-Proline^b^	18.1 (1.4)	16.7 (1.6)	0.004	0.02	7.5 (2.2)	9.0 (1.8)	0.02	< 0.0001
	*S*-Adenosyl-methionine	92.5 (30.4)	95.5 (26.9)	0.002	0.07	96.7 (31.9)	103.0 (33.7)	0.003	0.02

^a^All in µM except for *S*-Adenosylmethionine in nM.

^b^Log transformed variables with back-transformed means and standard deviations.

In children, methionine, tyrosine, proline, histidine, arginine, aspartic acid, glutamic acid, taurine, 1-methylhistidine, and *S*-adenosylmethionine plasma concentrations were not significantly different between males and females (Table 4). In adults, threonine, histidine, aspartic acid, and taurine plasma concentrations were not significantly different between males and females (Table 4).

## Discussion

Our study identifies family, sex, and age as important factors that characterise plasma AA concentrations. Both essential and non-essential AAs exhibited familial concordance in our study. The familial concordance of both essential and non-essential AAs supports a gene-environment contribution to AA profiles.

It has previously been demonstrated that non-essential AA concentrations exhibit a stronger concordance within an individual over time compared to essential AAs, which was proposed to be due to endogenous contributors to these profiles (i.e. genes and gene expression) being more stable than dietary intakes^16,17^. In our study, a mix of essential and non-essential amino acids exhibited the highest family effects. AAs exhibiting the highest family effects (> 20% of variability explained) were 3-methylhistidine (41%), aspartic acid (35%), isoleucine (34%), proline (33%), phenylalanine (31%), methionine (27%), taurine (25%), leucine (24%) valine (24%), and tyrosine (22%). Two important AA families are represented within this list: branched chain AAs (i.e. valine, leucine, and isoleucine), and aromatic AAs (i.e. phenylalanine, and tyrosine)^9^. This is interesting as a single overnight fasting plasma measurement of these 5 AAs (out of a total of 61 metabolites) predicted the development of type 2 diabetes up to 12 years later, and significantly improved the fit of predictive models that included traditional risk factors^1^. Other studies support the relationship between these AAs and adverse metabolic outcomes^2,18,19^. Identifying family effects for biomarkers of disease risk raises the possibility of characterising early metabolic targets, particularly within high risk families^20,21^.

Plasma AA concentrations vary with age; essential AAs were all lower in children compared to adults, and only non-essential plasma AA concentrations were higher in children. This profile of AAs in children may reflect an increased turnover of non-essential AAs and/or higher uptakes of essential AAs into peripheral tissues during anabolic growth phases in childhood, as previously postulated^22^. In the adult subgroup (28–71 years), the concentrations of 1-methylhistidine, citrulline, glutamic acid, glutamine, phenylalanine, and tyrosine were all weakly positively associated with increased age. Only threonine was weakly negatively associated with age. The weak association between these AAs and age may partly be explained by a non-homogenous distribution of adults in the 28–71 years age range. Age-specific differences in AA plasma concentrations may reflect an age-specific hormonal (e.g. insulin) regulation of AA uptake into peripheral tissues (e.g. muscles), where these AAs are utilised^23^. Decreased insulin sensitivity and lower lean body mass are characteristic of aging^24,25^, and the ratio of AA clearance in response to insulin has been demonstrated to be higher in younger compared to older adults^24^. Age is an important contributor characterising AA profiles in paediatric and adult populations^26,27^, and should be accounted for when interpreting AA concentrations.

We observed sex dependent changes in AA profiles, in agreement with published studies^7,22^. Sex specificity was more pronounced in adults than children with most AAs being higher in males, consistent with previous observations^28,29^. This may be explained by hormonal changes in early puberty (11–12 years) versus post-menarche/menopause (adulthood), affecting the concentrations of AAs. Moreover, there may be some age-dependent ‘maturation’ of physiological mechanisms involved in AA metabolism and regulation^16^. Factors underlying the age-specificity of AA profiles (i.e. insulin concentrations and lean body mass) are also sex-specific^24,25,30–32^. Females (a) have lower lean body mass compared with males^25,30^; and (b) exhibit higher glucose-mediated insulin sensitivity^24^, as well as (c) higher insulin secretion in response to the same blood glucose level as males^31^. Higher circulating insulin concentrations coupled with higher insulin sensitivity may further explain lower circulating AA concentrations in females, mediated by increased AA tissue uptake.

### Limitations

This was a large population based cross-sectional study in which plasma samples were only collected at a single timepoint. Extrapolations need to be drawn from our results carefully given that (a) our adults were mostly parents; (b) the sex distribution in the adult subgroup was unbalanced: A 1:10 male to female ratio in our cohort versus a ratio of 1:1 in the wider Australian population^33^; and that (c) the CheckPoint cohort comprised of socio-economically advantaged Australians: when averaging the top 3 SEIFA scores across Australian states, over 78% of our cohort scored in the middle to most advantaged socio-economic indexes for areas (SEIFA) compared to only 62% of the general Australian population^34^. Our study was also limited because we did not collect post-prandial samples, and all participants were semi-fasted (~ 4 h fast) at the time of blood collection. However, given that this was a systematic limitation across the entire population, and that fasting time was typically short and had a narrow distribution (children (4.4 ± 2.1 h [Mean ± SD]; Quartiles [Q1, Median; Q3] [3.38 h; 4.08 h; 4.85 h]) and adults (3.4 ± 2.4 [Mean ± SD]; Quartiles [2.19; 2.97; 3.94]), comparisons of relative amino acid concentrations would not have been largely impacted by baseline fasting status. Comparing AA concentrations across fasted and post-prandial states may have clarified the relative contribution of exogenous versus endogenous factors to AA profiles. Moreover, accounting for physical activity status/body composition, as well as anabolic and catabolic hormone concentrations by sex, age, and family relatedness could have further strengthened some of our conclusions. Finally, measures of insulin concentrations and/or sensitivity were not collected from our participants, although these may have informed on the underlying mechanisms of the sex and age specificity of AA concentrations.

## Conclusion

In this study, we identified a moderate concordance between children and parents from the same family for essential (diet-derived), and non-essential (diet derived and endogenously produced) AAs, as well as AA derivatives. This highlights a likely gene-environment behavioral contribution to circulating AA concentrations. The strongest familial concordance was evident for branched chain and aromatic AAs, which have been previously reported as strong predictors of diabetes mellitus, and have been also shown to be markedly associated with adverse metabolic outcomes^1,2^. We also identified age and sex-specific differences in AA profiles, that we suggest are partly attributable to age and sex-specific differences in lean body mass and insulin secretion/sensitivity.

## Supplementary Information

Supplementary Information.

## Data Availability

Data described in the article will be made available upon request after application and approval by our teams.

## Abbreviations

AA: Amino acid
BSA: Bovine serum albumin
EDTA: Ethylenediaminetetraacetic acid
LOQ: Limit of quantitation
MeOH: Methanol
MS–MS: Tandem mass spectrometry
OH-Proline: Hydroxyproline
PBS: Phosphate-buffered saline
SAH: S-Adenosylhomocysteine
SAM: S-Adenosylmethionine
TCEP: Tris(2-carboxyethyl)phosphine
UHPLC: Ultra-high-performance liquid chromatography
QC: Quality control
