# Supplementary material for: Population epidemiology and concordance for plasma amino acids and precursors in 11–12-year-old children and their parents
Source: Sci Rep. 2021 Feb 11;11:3619. doi: 10.1038/s41598-020-80923-9 (PMC7878730; doi:10.1038/s41598-020-80923-9)
Supplement: Supplementary file 1 — Supplementary Information. [file 41598_2020_80923_MOESM1_ESM.pdf]

Population epidemiology and concordance for plasma amino acids and precursors in 11-12-year-old children and their parents

Stephanie Andraos, Katherine Lange, Susan A. Clifford, Beatrix Jones,

Eric B. Thorstensen, Melissa Wake, David P. Burgner, Richard Saffery, Justin M. O'Sullivan

Supplementary data

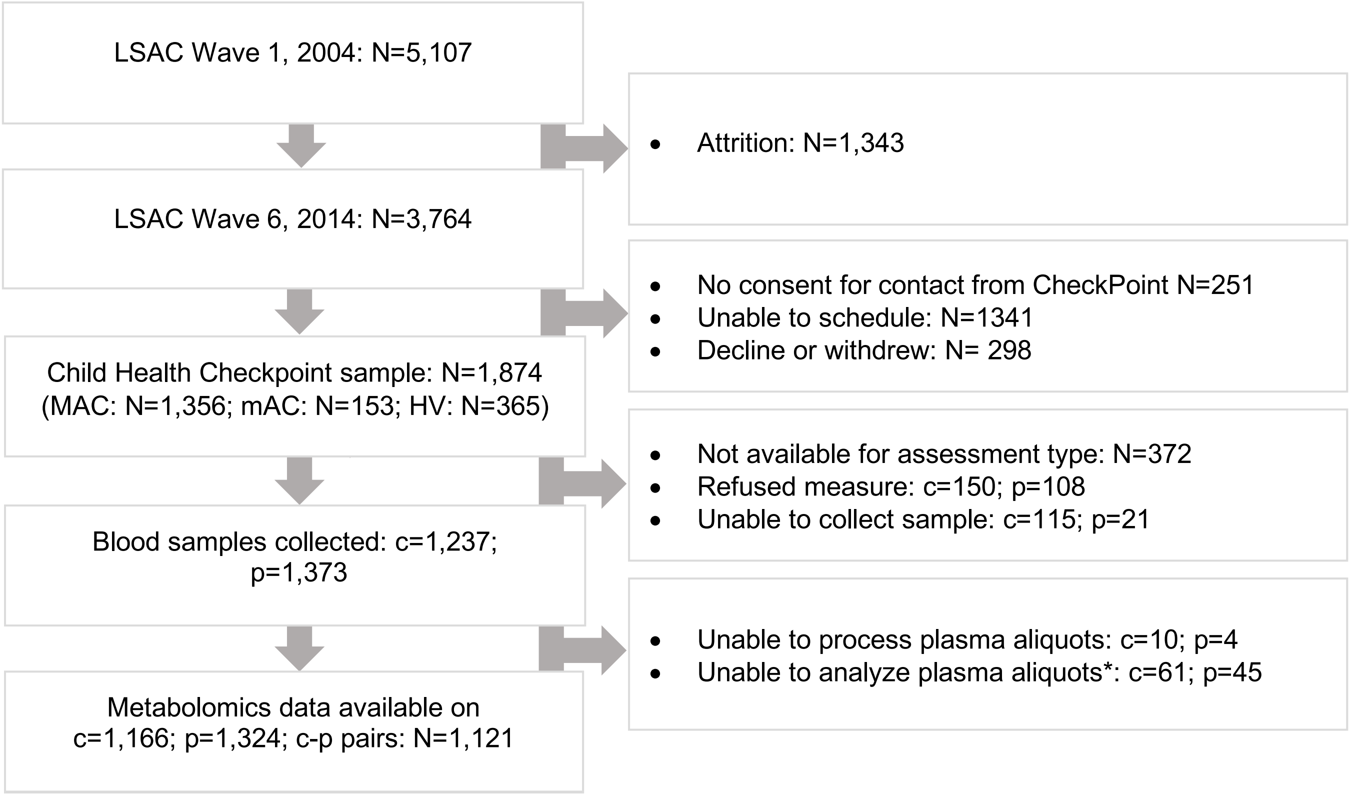

Supplementary Figure 1: Participant flow chart.

*Unable to analyse due to insufficient volume or poor-quality sample. HV: home visit; LSAC: Longitudinal Study of Australian Children; MAC: main assessment centre; mAC: mini assessment centre; N: number of families; p: number of attending adults; c: number of

Supplementary data

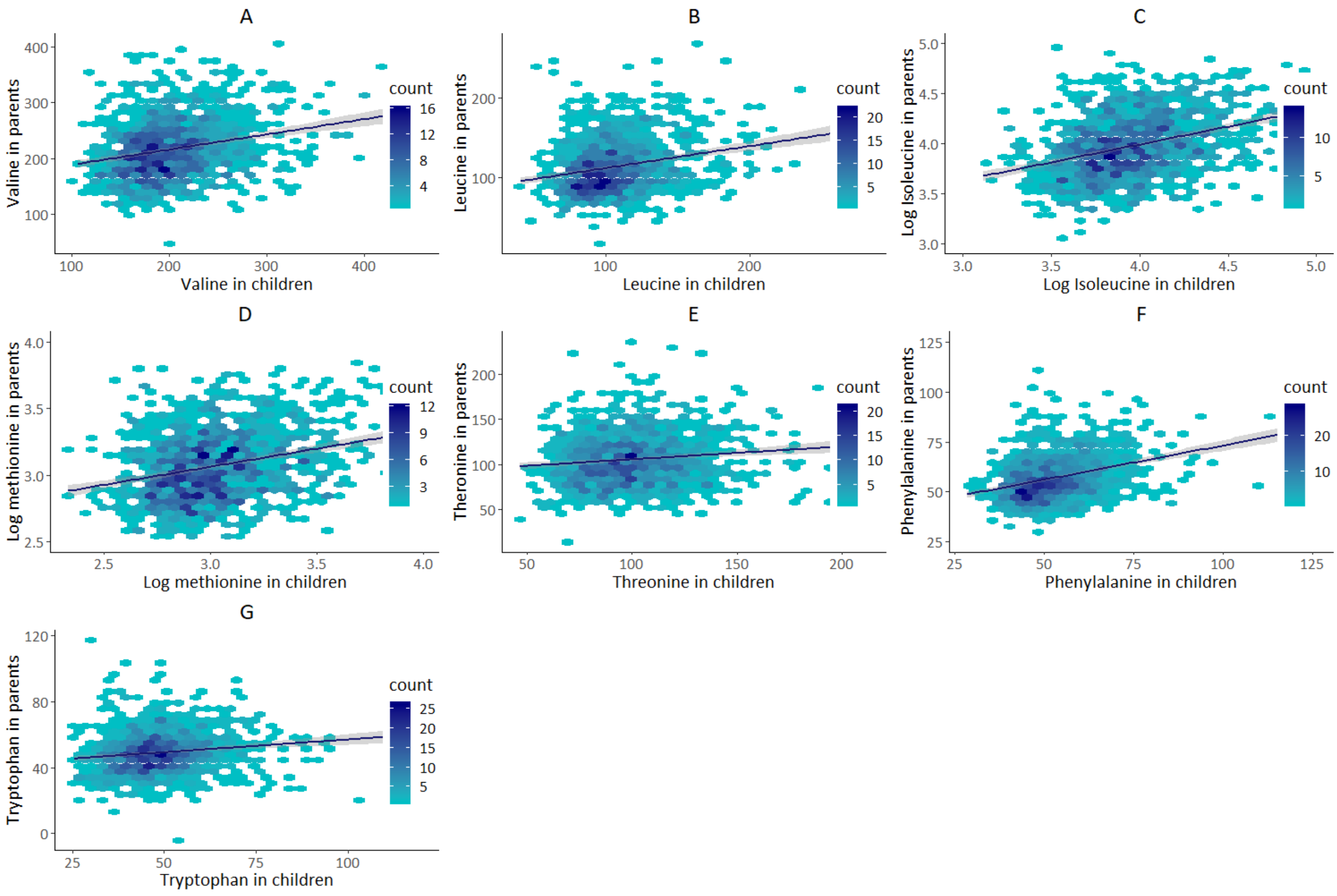

Supplementary Figure 2: Hexagonal plots of parent-child correlations for Valine (A), Leucine (B), Isoleucine (C), Methionine (D), Threonine (E), Phenylalanine (F), and Tryptophan (G)

Supplementary data

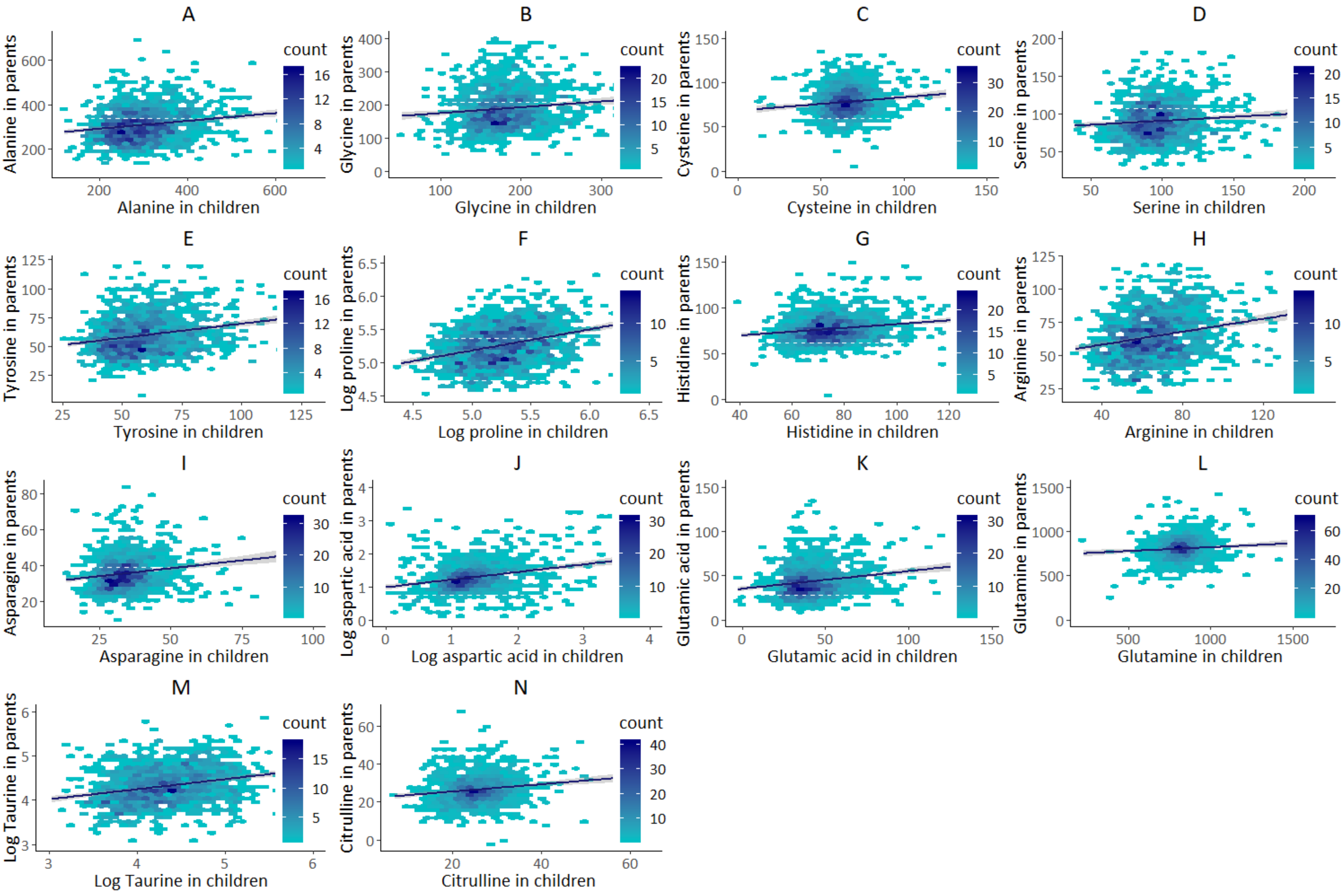

Supplementary Figure 3: Hexagonal plots of parent-child correlations for Alanine (A), Glycine (B), Cysteine (C), Serine (D), Tyrosine (E), Proline (F), Histidine (G), Arginine (H), Asparagine (I), Aspartic Acid (J), Glutamine (K), Glutamic Acid (L), Taurine (M), and Citrulline (N)

Supplementary data

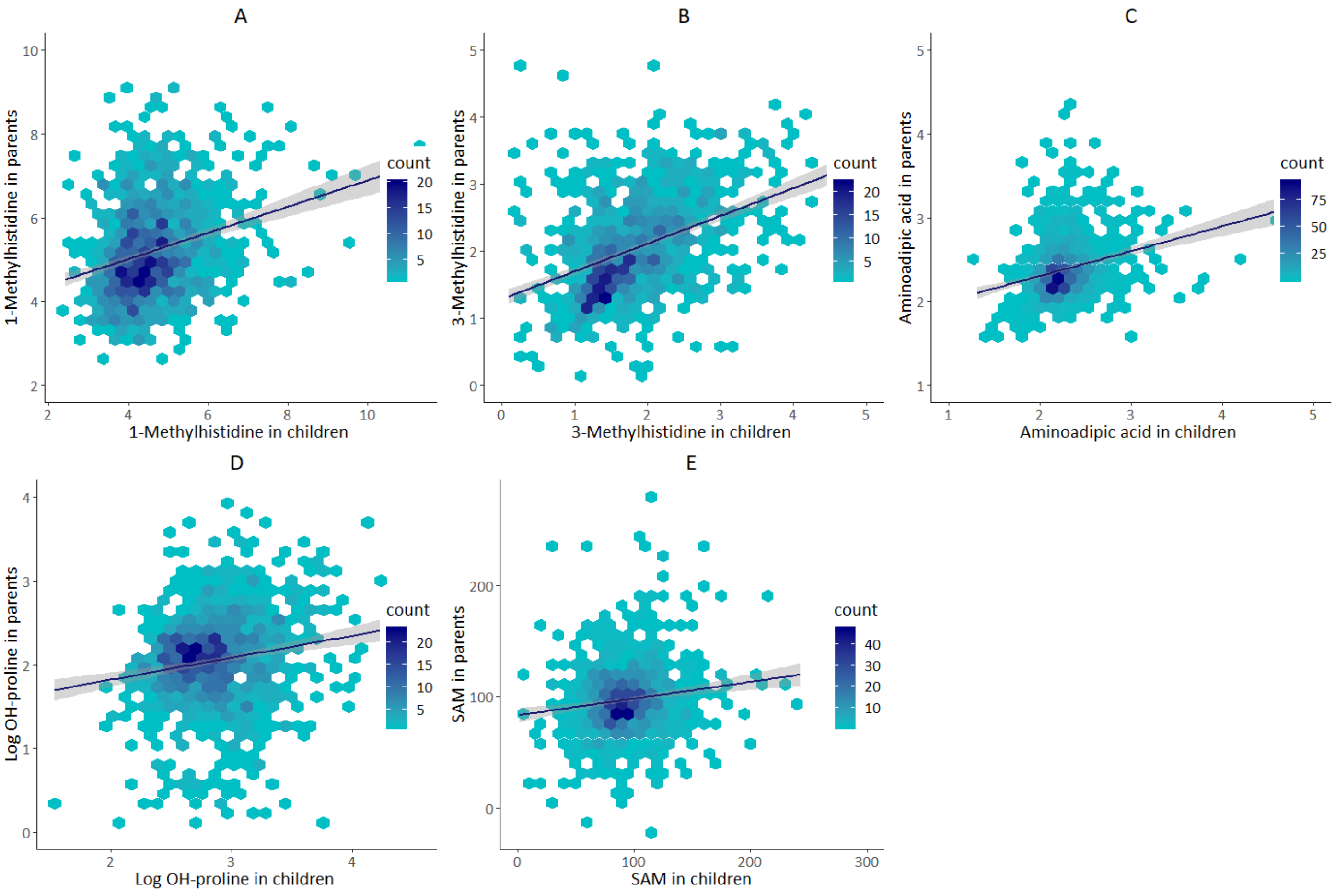

Supplementary Figure 4: Hexagonal plots of parent-child correlations for 1−Methylhistidine (A), 3−Methylhistidine (B), Aminoadipic Acid (C), OH−Proline (D), S−Adenosylmethionine (E)

Supplementary data

Supplementary Table 1:<br>Linear models of age-specific differences in the parent subgroup			
**Amino Acid***	**Compound Estimate**	**Adjusted R^2 of linear model**	**p value**
**Histidine**	-0.19	0.003	0.03
**1-Methylhistidine**	0.02	0.01	0.003
**3-Methylhistidine$^+$**	0.01	0.001	0.09
**Alanine**	-0.38	-0.0001	0.36
**Aminoadipic Acid**	0.0004	-0.001	0.91
**Arginine**	0.13	0.0004	0.22
**Asparagine**	-0.07	0.001	0.14
**Aspartic Acid$^+$**	-0.002	-0.001	0.56
**Citrulline**	0.26	0.03	<0.0001
**Glutamic acid**	0.25	0.005	0.005
**Glutamine**	2.44	0.01	0.0002
**Glycine**	-0.57	0.001	0.13
**Cysteine**	0.43	0.02	<0.0001
**Isoleucine$^+$**	0.002	0.0004	0.21
**Leucine**	0.21	0.0003	0.23
**Valine**	0.51	0.002	0.07
**Methionine$^+$**	0.003	0.002	0.08
**OH-Proline$^+$**	-0.004	0.0002	0.27
**Proline$^+$**	0.001	-0.0004	0.47
**Phenylalanine**	0.16	0.004	0.01
**S-Adenosylmethionine**	0.83	0.02	<0.0001
**Serine**	-0.15	0.0003	0.24

**Threonine**	-0.31	0.002	0.05
**Tryptophan**	0.09	0.0005	0.20
**Tyrosine**	0.30	0.008	0.001
**Taurine⁺**	0.0004	-0.001	0.86

** All in µM except for S-Adenosylmethionine in nM*

⁺ Log transformed variables with back-transformed means and standard deviations
